# Three-Dimensional Magneto-Optical Trap Beam Delivery with Scalable Wafer-Level Optics

**DOI:** 10.1021/acsphotonics.6c00337

**Published:** 2026-04-29

**Authors:** Zi Wang, Phillip S Cloud, Minsuk Lee, Wenqi Zhu, Kanghee Won, Qian Sun, Amit Agrawal

**Affiliations:** † 10833 National Institute of Standards and Technology, Gaithersburg, Maryland 20899, United States; ‡ University of Maryland, College Park, Maryland 20742, United States; § Key Laboratory of Semiconductor Display Materials and Chips, 85404 Suzhou Institute of Nano-Tech and Nano-Bionics, Chinese Academy of Sciences, Suzhou 215123, P. R. China; ∥ Department of Engineering, 2152 University of Cambridge, Cambridge CB3 0FA, U.K.; ⊥ 26723 Kyung Hee University, 26 Kyungheedae-ro, Dongdaemun-gu, Seoul 02447, Korea

**Keywords:** metagrating, inverse design, magneto-optical trap, photonic integrated circuit, topology optimization

## Abstract

Magneto-optical traps (MOTs) are essential for preparing and capturing cold atoms in precision atomic applications. Conventional free-space beam delivery systems are bulky, expensive, and difficult to align. Current photonic integrated circuit (PIC) MOT approaches, although scalable, still require manual alignment of external quarter-wave plates (QWPs) and mirrors, limiting miniaturization and reduction of complexity. Here, we present a two-wafer beam delivery architecture for a PIC-based MOT using an inverse-designed multifunctional grating coupler and integrated metasurface retroreflectors, eliminating the need for all external optics. Both components are designed via inverse optimization to deliver circularly polarized light for ^87^Rb at 780 nm. We experimentally demonstrate a retroreflector with 74% efficiency and 0.85 ellipticity, meeting performance requirements for MOT operation. The grating coupler is characterized through full-wave electromagnetic simulations, showing 20% out-coupling efficiency and 0.85 polarization ellipticity. This two-wafer architecture proposed here significantly simplifies alignment and maintains a compact, scalable, foundry-compatible footprint for all beam-delivery components.

## Graphical abstract

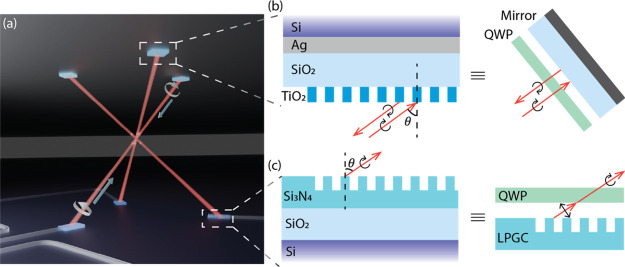

## Introduction

Quantum systems based on cold atoms form the backbone of a wide array of critical technologies, including precision spectroscopy,^1^ optical atomic clocks,^2,3^ and advanced sensing.^4^ Among different technologies, the magneto-optical trap (MOT), which combines magnetic fields and laser beams to efficiently cool and trap large populations of atoms near absolute zero, has become one of the most important tools. A standard MOT is formed by three orthogonal pairs of counter-propagating, circularly polarized laser beams intersecting at the zero-point of a quadrupole magnetic field generated by anti-Helmholtz coils.^5^ As atoms move away from the trap center, the spatially varying magnetic field induces a position-dependent Zeeman shift in their energy levels, bringing them into resonance with the opposing laser beam. This creates a spatial restoring force that, in combination with the velocity-dependent damping of Doppler cooling, continuously cools and traps the atoms. However, this conventional six-beam and dual-coil configuration typically results in a bulky and complex structure. To reduce the size-weight-and-power (SWaP) of the MOT system, different approaches have been proposed, including grating MOT (GMOT),^6–11^ Fresnel MOT,^12^ metasurface MOT,^13–18^ and photonic integrated circuit (PIC) MOT.^19,20^ The GMOT utilizes a set of diffractive gratings to generate three or more beams from a single incident beam, enabling significant system miniaturization. Like PICs and metasurfaces, microfabricated GMOTs benefit from scalable, wafer-level planar manufacturing processes. However, because GMOTs sacrifice the high degree of symmetry of a standard six-beam MOT, their performance is usually worse than that of the conventional setup. Recently, the Fresnel MOT has emerged as another promising single-beam architecture, utilizing a Fresnel reflector to achieve atom numbers competitive with six-beam setups while maintaining a compact form factor.^12^ Alternatively, metasurface MOTs and PIC MOTs can form a highly symmetric, standard six-beam MOT with a reduced SWaP. Both metasurfaces and PICs have the advantage of being compatible with complementary metal oxide semiconductor (CMOS) fabrication, which reduces manufacturing costs and enables scalability. A metasurface MOT can easily generate beams with the required directionality and polarization; however, it still requires external optics to independently route the incident laser beams, demanding precise optical alignment and complex assembly procedures. On the other hand, PIC MOTs have the potential of being fully integrated with on-chip lasers via heterogeneous integration but still require external bulk photonic components, including waveplates and mirrors. By combining inverse-designed metasurface optics with PICs, we achieve precise control of beam direction and polarization with the potential for heterogeneous integration. Here, we propose a system combining metasurfaces and PICs to form a MOT by using multifunctional grating couplers to couple wave-guided laser beams out of the plane and convert their polarization to circular and novel retroreflectors, which maintain handedness upon reflection to reflect circularly polarized laser beams. These two inverse-designed components form the basis of a six-beam MOT architecture and offer the potential for planar integration, foundry manufacturability, and significant reduction of the overall SWaP of the system.

Traditional methods based on forward design for both metasurface and grating coupler implementations cannot achieve the desired multifunctionality of simultaneous control of polarization and beam directionality. In order to achieve the desired functionality from a single-layer optic while maintaining high efficiency, here we use an inverse design method based on a reinforcement learning algorithm. The proposed metasurface retroreflector and a PIC grating coupler are designed to operate at λ = 780 nm to address the D_2_ transition in ^87^Rb for the realization of a 3D-MOT (Figure 1a). Both retroreflector and grating coupler are optimized respectively to reflect or generate circularly polarized light with an operating angle of θ = 54.7° and placed 120° apart from each other to cover three orthogonal directions required for a MOT (Figure 1a). With freeform inverse design, we can design a metagrating reflector in a Littrow configuration with a reflection layer on the bottom, which functions as a tilted mirror with a quarter-wave plate (QWP) on top of it (Figure 1b). We also design a freeform grating coupler with inverse design such that the out-coupled light is circularly polarized. Such a grating coupler functions as a regular linearly polarized grating coupler (LPGC) with a QWP on top (Figure 1c). With these devices, beam delivery of a 3D-MOT can be achieved with two wafers without relying on any bulk components (Figure 1a), which makes the entire beam-delivery system CMOS foundry compatible, greatly reducing the cost, complexity, and SWaP while improving the scalability, robustness, and manufacturability.

**Figure 1:**
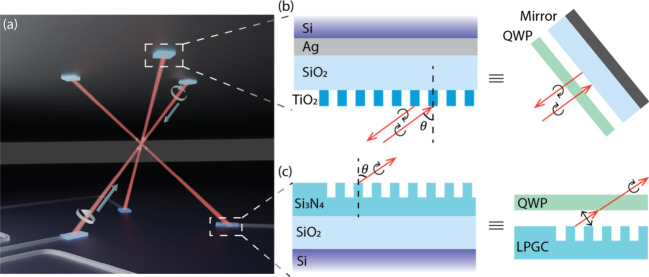
Schematic of (a) proposed planar two-wafer magneto-optical trap system. (b) Polarization-maintaining retroreflector, shown along with its equivalent bulk-optics embodiment which consists of a mirror and a quarter waveplate (QWP) mounted orthogonal to the beam propagation axis. (c) Integrated photonic circularly polarized grating coupler, shown along with its traditional PIC equivalent, which consists of a linear polarized grating coupler and a QWP.

## Results and Discussion

### Inverse Design Algorithm

Over the past decade, different freeform design methods for inverse design of photonics devices have been proposed.^21–25^ Unlike traditional design methods, which are limited to fixed shapes, freeform design allows topology variations with higher degrees of freedom.^21^ There are different algorithms for freeform inverse design, such as adjoint-based and deep-learning-based methods. The adjoint-based method determines the gradient of a figure of merit (FoM) with respect to design parameters by performing a single forward and a single backward simulation, and this gradient is then used to iteratively converge toward a local optimum.^23,24^ Such a local optimum solution is usually suboptimal as it is strongly influenced by the initial guess of the design parameters. In contrast, deep learning offers a data-driven approach that can handle complex design objectives by exploring a vast parameter space without the need for a high-quality initial guess.^21,25^ Within the realm of deep learning, reinforcement learning (RL) eliminates the need for pre-existing data sets.^21–24^ Instead, the neural network begins in a random state and iteratively discovers optimal solutions by rewarding desired behaviors and penalizing undesired ones. Within the RL framework, various algorithms have been developed to suit specific applications, including Deep Q-learning (DQN),^26^ Proximal Policy Optimization (PPO),^27^ Deep Deterministic Policy Gradient (DDPG),^28^ and Soft Actor-Critic (SAC).^29^ The inverse design algorithm we used here is based on SAC, which is an interpolation of DQN and PPO. There are two neural networks in SAC: the actor network π and critic network *Q*. The actor network π is used to generate a refractive index distribution pattern of the device, and the critic network *Q* is used to predict the reward, which is related to the target optical function of the device. After training, the SAC algorithm predicts the reward accurately and generates patterns with maximized rewards. The algorithm utilizes a Fourier transform to generate a pattern with a smaller matrix, and a morphological filter (MF) is applied next to enforce fabrication constraints. A rigorous coupled-wave analysis (RCWA) simulation is performed to evaluate the generated pattern. The details of the algorithm can be found in a previous study.^30^

### Polarization Maintaining Retroreflector

A retroreflector can be achieved by making a reflective diffraction grating in a Littrow configuration, in which the diffraction angle and incidence angle are identical. Thus, the diffracted beam is back-reflected in the direction of the incident beam. Such a configuration is governed by the grating equation:

$$\Lambda [\sin(\theta_{\mathrm{in}})+\sin(\theta_{\mathrm{out}})]=m\lambda$$ 1

where Λ is the period of the grating, θ_in_ and θ_out_ are the incident and diffracted light, *m* is the diffraction order, and λ is the wavelength of light. When in Littrow configuration, θ_in_ is equal to θ_out_, and eq 1 becomes 2Λ­[ sin (θ)] = *m*λ. So, the Littrow angle is dependent on the period of the grating, the wavelength of light, and the diffraction order. A regular grating works only for transverse electric (TE) or transverse magnetic (TM) polarization, making it unsuitable for working with circularly polarized light. Here, we demonstrate a retroreflector for circularly polarized light with a freeform metagrating in Littrow configuration, as shown in Figure 2a. We use the reinforcement learning-based inverse design algorithm mentioned earlier to design the metagrating. We first determine the period of the grating to be Λ_*x*_ = λ/[2·sin (θ)], and Λ_*y*_ = λ/2 (Figure 2b), where λ = 780 nm, and θ = 54.7°. The thicknesses of TiO_2_, SiO_2_, and Ag are optimized to *T*_TiO_2__ = 440 nm, *T*_SiO_2__ = 330 nm, and *T*_Ag_ = 100 nm, respectively. These values were determined through parameter sweeping to maximize the reflectivity. The target function to maximize during the inverse design optimization is set as

$$r=\left(\frac{b}{a}\right)\cdot \frac{\left|E_{\mathrm{out}}^{s}\cdot e^{j\Delta \phi_{\mathrm{out}}}+E_{\mathrm{out}}^{p}\cdot e^{j\Delta \phi_{d}}\right|}{\sqrt{2\cdot (|E_{\mathrm{in}}^{s}{|}^{2}+|E_{\mathrm{in}}^{p}{|}^{2})}}$$ 2

where *a* and *b* are the lengths of the major and minor axes of the output polarization ellipse, *E*_in_^s^ (*E*_out_^s^) and *E*_in_^p^ (*E*_out_^p^) are the s-polarized (*E*-field perpendicular to the *x-z* plane) and p-polarized (*E*-field parallel to the *x-z* plane) electric field of the input (output) light propagating at an angle of θ with respect to the *z-*axis, respectively, and Δϕ_d_ is the desired phase difference between *E*_out_^s^ and *E*_out_^p^, which should be $\pm \frac{\pi }{2}$ for circularly polarized light. For the perfect circularly polarized output beam with a reflectivity of 100%, the value of the target function is 1, and we maximize the target function in the inverse design process to generate devices with high reflectivity and ellipticity. The reflectivity is defined as the power of the reflected beam divided by the power of the incident beam, $\eta =\frac{P_{\mathrm{refl}}}{P_{\mathrm{inc}}}$ . The ellipticity is defined as the ratio *b*/*a*, where *b* is the length of the minor axis, and *a* is the length of the major axis of the polarization ellipse. Based on the experimentally measured refractive index of TiO_2_ at 780 nm (2.352), we performed simulations using rigorous coupled-wave analysis (RCWA) and full 3D finite difference time domain (FDTD). Using FDTD simulations, we calculate a reflectivity of 98.4% and an ellipticity of 0.88 for the optimized devices.

**Figure 2:**
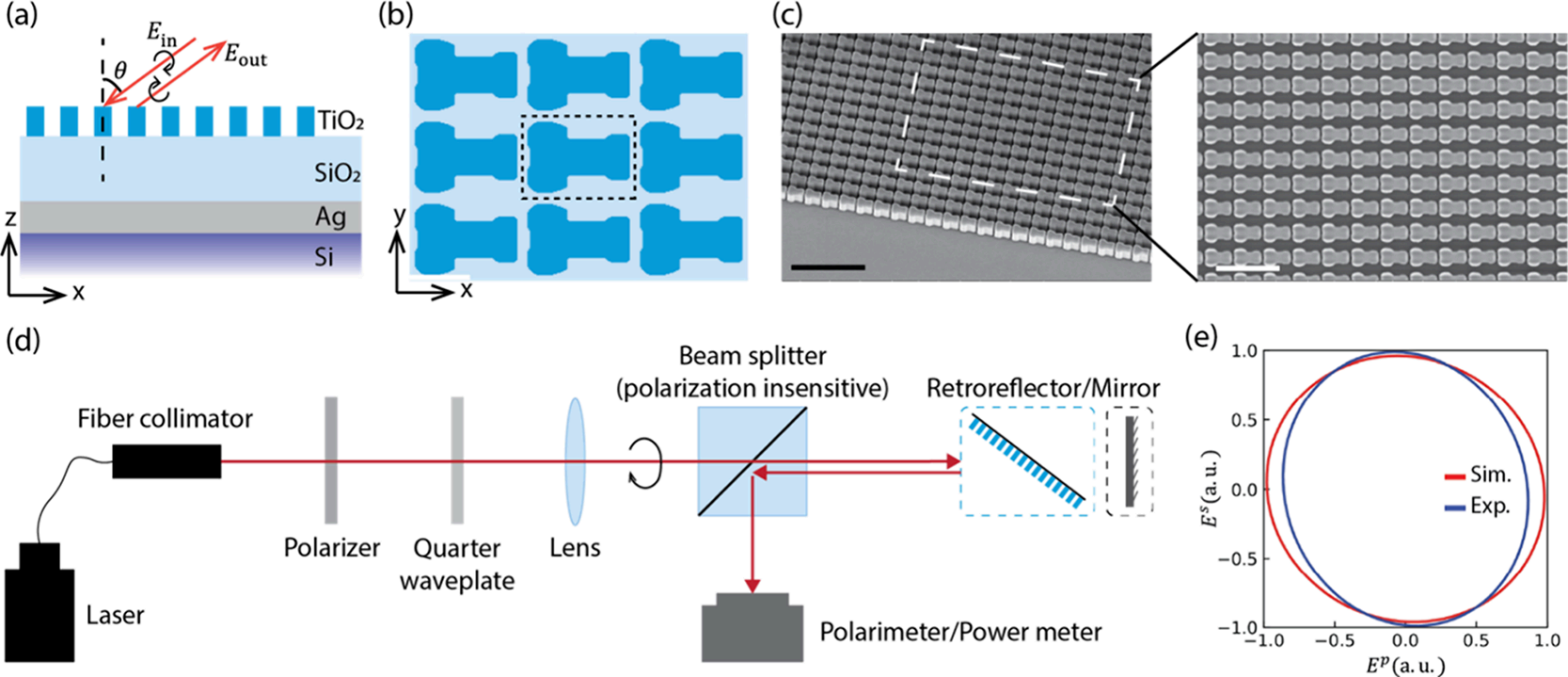
Polarization-maintaining retroreflector. (a) Schematic side view of the retroreflector. Retroreflectors are made with TiO_2_ metagrating on the top of a SiO_2_ spacing layer and Ag reflective layer on a Si substrate. The incident and output angles, defined as the angles from the normal of the *x-y* plane to the ray directions, are positive if measured clockwise and negative if measured counterclockwise. (b) Schematic top view of the retroreflector, and the unit cell is shown in the dashed box. (c) Tilted and top view SEM images of the fabricated retroreflector. The scale bars are 2 μm (left) and 1 μm (right). (d) Schematic of the experiment setup for measuring polarization ellipticity and power of the beam reflected by the retroreflector or a reference mirror. (e) Simulated and experimentally measured polarization ellipses of the output electric field.

Using these optimized parameters, we fabricated the retroreflector devices using sputter deposition, electron beam lithography (EBL), and the damascene process as described in a previous study.^31^ The Ag film with a nominal thickness of *T*_Ag_ = 100 nm was deposited using sputtering, immediately followed by deposition of a SiO_2_ film with a nominal thickness of *T*_SiO_2__ = 330 nm. The sample is then coated with a 440 nm thick positive-tone electron beam resist (ZEP 520A). Then, the pattern of the metagrating was defined on the resist using EBL with 100 kV accelerating voltage and 0.5 nA beam current. Next, the resist was developed in hexyl acetate developer at 4 °C, and then the pattern was filled by depositing TiO_2_ using atomic layer deposition. Next, the overcoated TiO_2_ layer was etched to the resist top surface by inductively coupled plasma reactive ion etching, and the residual resist was removed by being soaked in n-methyl-2-pyrrolidone. The fabricated film thicknesses were characterized individually using a spectroscopic ellipsometer, and the device topography was measured using a scanning electron microscope (SEM). The SEM images of the fabricated devices show good reproducibility and a close match with the design parameters (Figure 2c). The fabricated devices were experimentally characterized by using a custom-built optics setup (Figure 2d). A circularly polarized beam at λ = 780 nm was used to illuminate the sample at θ = 54.7°. In the experiment, the efficiency is defined as $\eta_{m}=\frac{P_{\mathrm{retro}}}{P_{\mathrm{mirror}}}$ , where *P*_retro_ is the measured power of the beam reflected by the retroreflector, and *P*_mirror_ is the measured power of the beam reflected by a reference mirror. We used a lens with a focal length of 250 mm to gently focus the light and ensure all input beams pass through the retroreflector. The polarization state was measured using a calibrated polarimeter, and power was measured using a power meter. The experimentally measured ellipticity of the beam is 0.85, and the measured efficiency is 74%. The small discrepancy between the experimentally measured and simulated values (Figure 2e) stems from the strong dependence of the thickness of the SiO_2_ layer on the device performance, and slight discrepancies between optimized and fabricated device dimensions.

### Circular Polarization Grating Coupler

Using the same inverse design algorithm, we design grating couplers on a shallow (150 nm deep) etched Si_3_N_4_ layer (250 nm thick) with 500 nm SiO_2_ top cladding to control the properties of the out-coupled beam from the waveguide plane. To accurately control the out-of-plane beam, we model the light propagation in the grating coupler region, as shown in Figure 3a. The grating coupler is composed of a periodic metagrating, and the beam propagation also follows the grating equation. After simulation and calculation, we determine the period of the metagrating as Λ_*x*_ = 313.7 nm and Λ_*y*_ = 390 nm. We then perform the inverse design to decide the structure of the metagrating cell, and the top view of the metagrating is shown in Figure 3b. As shown in Figure 3a, once in the waveguide structure, the beam reflected by the back side of the waveguide becomes the input beam again. Because of the back reflection by the waveguide structure, the target function in the inverse design is different from the free-space metagrating design. The target function for grating coupler inverse design is

$$r=(R+T)\cdot \left(\frac{b}{a}\right)\cdot \frac{\left|E_{\mathrm{out}}^{s}\cdot e^{j\Delta \phi_{\mathrm{out}}}+E_{\mathrm{out}}^{p}\cdot e^{j\Delta \phi_{d}}\right|}{\sqrt{2\cdot (|E_{\mathrm{out}}^{s}{|}^{2}+|E_{\mathrm{out}}^{p}{|}^{2})}}$$ 3

where *a* and *b* are the major and minor axes of the output polarization ellipse, respectively, Δϕ_d_ is the desired phase difference between the output electric fields *E*_out_^s^ and *E*_out_^p^, *R* is the reflectance of light with angle of reflection θ_r_ = θ_in_, and *T* is the transmittance of the order with θ_out_ = −54.7°. For circularly polarized light, the value of $\Delta \phi_{d}=\pm \frac{\pi }{2}$ , and *a* = *b*. After inverse design optimization, we first verify the designed metagrating using a free-space 3D FDTD simulation. In free space simulation, we simulate one metagrating cell with periodic boundary conditions, as shown in the black box in Figure 1a. The incident angle is θ_in_ = 56.7°, and the output angle is θ_out_ = −54.7°. The simulated ellipticity of the polarization ellipse is 0.82. After the free-space FDTD simulation, we performed an FDTD simulation in the grating coupler configuration. The input TE mode is imported by a mode source in FDTD, and we place a monitor above the top SiO_2_ cladding layer. The far-field intensity distribution of the out-coupled beam is plotted in Figure 3c, and the out-coupled angle is calculated to be −54.7°, the same as the design. The coupling efficiency is measured to be 20%, and the simulated ellipticity of the out-coupled beam at θ_out_ = −54.7° is 0.85, as shown in Figure 3d.

**Figure 3:**
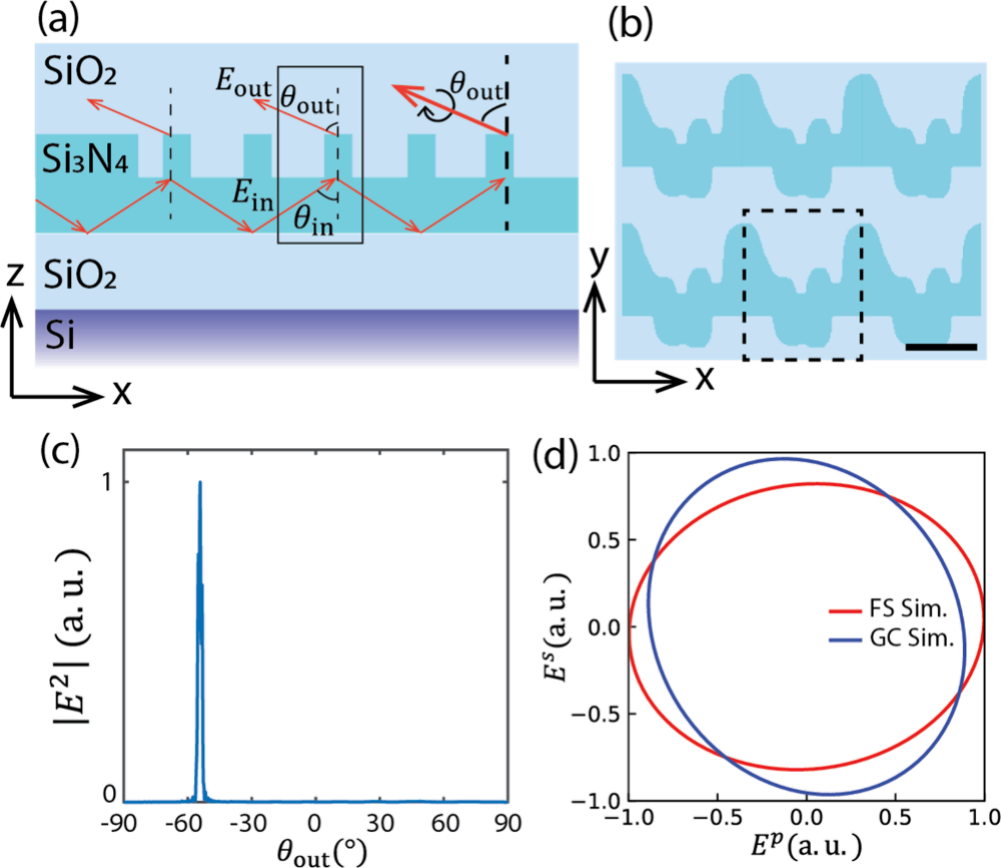
Integrated photonic grating coupler for circularly polarized light. (a) Schematic side view of the grating coupler. (b) Schematic top view of the designed grating coupler, and the unit cell is shown in the dashed box. Scale bar, 200 nm. (c) FDTD simulated far-field intensity distribution versus the output angle, and the grating coupler is designed for θ_out_ = −54.7°. (d) Simulated polarization ellipses of the out-coupled light in two different simulation configurations: free space periodic simulation and integrated photonic grating coupler simulation.

### Proposed 2-Wafer System Integration

The proposed system can be constructed by placing two wafers parallel and facing each other, separated by a distance *d*, as shown in Figure 4a. In each wafer, three gratings are placed 120° apart from each other, and on a circle with radius *r*_1_ and *r*_2_, respectively, for grating couplers and retroreflectors. Three parameters, *d*, *r*_1_, *r*_2_, and out-coupled angle θ_out_, are interdependent, as shown in the equation *d* = (*r*_1_ + *r*_2_)·cot (θ_out_). The beam intersection position above the PIC wafer is calculated by *h* = *r*_1_·cot (θ_out_), where θ_out_ = 54.7° ensures that six beams are mutually orthogonal. The beam profile at 20 mm above the PIC chip is a Gaussian shape, as shown in Figure 4b.

**Figure 4:**
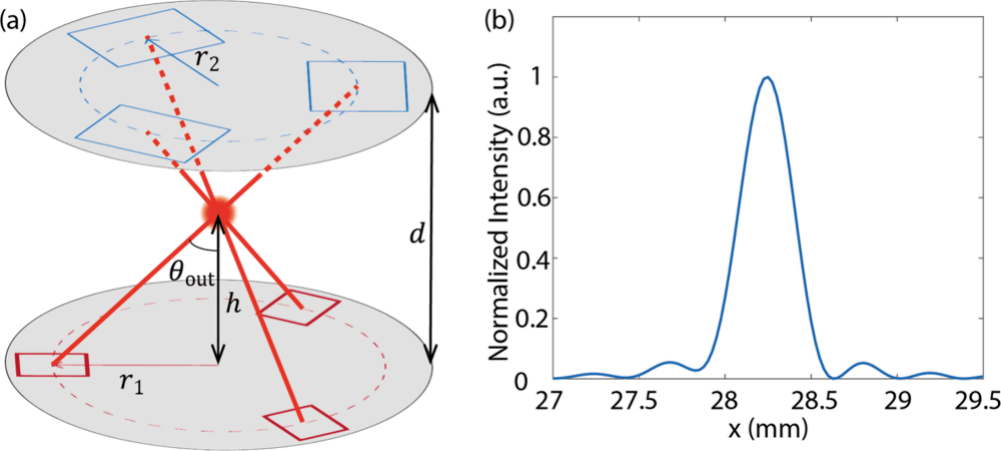
Integration of the 2-wafer system. (a) Schematic view of the proposed system. (b) Simulated far-field profile of the output beam from the grating coupler at *z* = 20 mm.

Achieving precise structural and optical alignment between the two opposing wafers is critical to the overall trapping performance of the system. We decouple this challenge into translational and angular components. In our proposed design, the requirements for exact translational alignment are heavily relaxed simply by increasing the active area of the retroreflector, which naturally accommodates minor lateral offsets. Angular alignment, encompassing both in-plane rotation and out-of-plane tilt, is handled passively. By utilizing the built-in wafer flats for azimuthal orientation and a precision-machined wafer holder to maintain exact parallelism, the system is designed to achieve robust alignment without the need for complex tuning. The experimental feasibility of this specific passive alignment technique has been rigorously verified in our prior work.^15^

The overall success of the system also relies on the performance of the individual components. Our calculated PIC efficiency of 20% is highly suitable for MOT operation, exceeding the 15% efficiency previously shown to be sufficient for atom trapping.^20^ Although the measured retroreflector efficiency is presently 74%, simulations predict an achievable efficiency of 98%, leaving significant room for improvement through careful optimization. Additionally, potential radiation force imbalances arising from unmatched beam intensities in the overlap region can be effectively mitigated. By altering the arrangement of the metagrating unit cells, the out-coupled beam can be made divergent or the reflected beam made convergent to balance the forces. The circular polarization retention (ellipticity ≥0.85) also meets requirements for efficient Doppler cooling and trapping, as detailed analysis shows minimal loss of trap depth with ellipticity >0.8.^32,33^

## Conclusion

In conclusion, this work demonstrates a scalable two-walled architecture for PIC-based MOT beam delivery, combining grating couplers and metasurface retroreflectors designed through inverse optimization. The retroreflector was experimentally validated, and the grating coupler performance is characterized through rigorous electromagnetic simulations. By eliminating external QWPs and mirrors, this approach significantly reduces the alignment complexity and system footprint compared to current PIC-MOT implementations. The design is compatible with foundry fabrication processes, enabling cost-effective scaling. Future work will demonstrate the full integrated system with experimental demonstration of atom trapping and characterization of MOT performance metrics (atom number, temperature, and loading time).

## Data Availability

Data underlying the results presented in this paper are not publicly available at this time but may be obtained from the authors upon reasonable request.
